# Evolutionary constraint on low elevation range expansion: Defense‐abiotic stress‐tolerance trade‐off in crosses of the ecological model *Boechera stricta*

**DOI:** 10.1002/ece3.5499

**Published:** 2019-10-02

**Authors:** Jason Olsen, Gunbharpur Singh Gill, Riston Haugen, Steven L. Matzner, Jake Alsdurf, David H. Siemens

**Affiliations:** ^1^ Integrative Genomics Program Black Hills State University Spearfish SD USA; ^2^ Biology Department Augustana University Sioux Falls SD USA; ^3^ Present address: 500 W Fort Street 111R Boise ID 83702 USA; ^4^ Present address: Department of Biology Utah State University Logan UT USA; ^5^ Present address: Division of Biology Kansas State University Manhattan KS USA

**Keywords:** abiotic stress‐tolerance, *Boechera stricta*, chemical defense, extended generation crosses, geographical range limits, linkage mapping, trade‐offs

## Abstract

Most transplant experiments across species geographic range boundaries indicate that adaptation to stressful environments outside the range is often constrained. However, the mechanisms of these constraints remain poorly understood. We used extended generation crosses from diverged high and low elevation populations. In experiments across low elevation range boundaries, there was selection on the parental lines for abiotic stress‐tolerance and resistance to herbivores. However, in support of a defense‐tolerance trade‐off, extended generation crosses showed nonindependent segregation of these traits in the laboratory across a drought‐stress gradient and in the field across the low elevation range boundary. Genotypic variation in a marker from a region of the genome containing a candidate gene (MYC2) was associated with change in the genetic trade‐off. Thus, using crosses and forward genetics, we found experimental genetic and molecular evidence for a pleiotropic trade‐off that could constrain the evolution of range expansion.

## Graphical abstract

Evolutionary constraints are important to understand range limits, yet the mechanisms are poorly understood. Here we used an integrative approach to show that a trade‐off between abiotic stress‐tolerance and chemical defense against herbivores may inhibit the evolution of low elevation range expansion in the ecological model *Boechera stricta*.

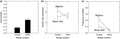

## 1 INTRODUCTION

Multiple natural phenomenon such as extinction, conserved phylogenetic niche evolution, and limits on geographical and habitat range indicates that constraints are important in the process of biological evolution in the wild. This is also supported by experimentation. At species range boundaries, for example, most transplant studies (75%) show decreased performance across range boundaries (Hargreaves, Samis, & Eckert, 2014; Lee‐Yaw et al., 2016; Sexton, McIntyre, Angert, & Rice, 2009 for reviews), indicating that adaption is often constrained. However, the mechanisms of these constraints are poorly understood (Futuyma, 2010). As global climate change forces natural experiments at species range boundaries, it will be important to understand evolutionary constraints to help predict local adaptation or extinction.

Several factors have been proposed that influence the process of adaptation to stressful environments found just across range boundaries or in new areas because of climate shifts, but evidence is limited (Sexton et al., 2009). Some possibilities include gene flow from elsewhere in the range (Sexton, Strauss, & Rice, 2011), lack of genetic variation within and among range margin populations (Eckert, Samis, & Lougheed, 2008), barriers to dispersal or establishment, and genetic, physiological, or developmental trade‐offs (Chuang & Peterson, 2016; Kawecki, 2008). Here, we test a novel hypothesis regarding trade‐offs inspired by signaling networks that may constrain simultaneous adaptations at some range boundaries.

At warm edge range boundaries, such as at low latitudes or low elevations, both abiotic and biotic stressors are more likely to be present and impactful compared to elsewhere in the range (Cahill et al., 2014; Ettinger, Ford, & Hillerislambers, 2011; Louthan, Doak, & Angert, 2015), but see Chesson and Huntly (1997). Therefore, at these lower boundaries, adaptation to both abiotic and biotic stressors simultaneously may be required (Anstett, Nunes, Baskett, & Kotanen, 2016 for review of predicted increase in defense at lower latitudes, Baskett, Schemske, & Novotny, 2018). However, in work with Arabidopsis and other plant species, plastic responses to the simultaneous challenges of abiotic and biotic stressors often result in trade‐offs (Asselbergh, Vieesschauwer, & Hofte, 2008; Atkinson & Urwin, 2012; Fujita et al., 2006; Ton, Flors, & Mauch‐Mani, 2009 for reviews). Drought‐stressed plants, for example, may have attenuated responses to subsequent challenges by biotic factors such as disease or herbivores. The molecular basis of these trade‐offs involves signaling pathways and genes of major effect, often transcription factors (TFs), which coregulate the pathways. Transcription factors can be major hubs in signaling networks that help coordinate simultaneous responses to multiple environmental challenges. Therefore, TFs might also be targets of selection at lower range boundaries.

Here we conducted experimental crosses and used a candidate gene linkage‐mapping approach to begin to evaluate the role of major effect TFs in constraining range limits. We used the perennial *Boechera stricta*, an ecological model (Rushworth, Song, Lee, & Mitchell‐Olds, 2011), close relative of *Arabidopsis thaliana*, and native to mountainous regions of western North America. We crossed high and low elevation populations that have diverged for an evolutionary trade‐off between abiotic stress‐tolerance and chemical defense against generalist insect herbivores. Neither population had high levels of both traits (Gill, Haugen, Larson, Olsen, & Siemens, 2016; Gill, Haugen, Matzner, Barakat, & Siemens, 2016).

The overarching hypothesis is therefore that selection acts on defense and abiotic stress‐tolerance to expand low elevation range boundaries, but pleiotropic factors such as the TFs in signaling pathways prevent the simultaneous evolution of these traits. Thus, using experimental genetics of extended generation crosses, the main questions of interest were as follows: (a) whether abiotic stress‐tolerance and defense against herbivores segregated independently of one another as predicted by a pleiotropic evolutionary trade‐off and (b) whether this pleiotropic factor occurred in the region of the genome containing a candidate TF that coregulates defense and stress‐tolerance signaling pathways. Specifically, to do this, we first (a) documented the abiotic stress‐tolerance and glucosinolate (GS) defense trait differences between the high and low elevation populations represented by the parental lines used in the crosses. In field experiments comparing high and low elevation populations, we then (b) determined whether there was selection for abiotic stress‐tolerance and resistance to herbivores across low elevation range boundaries as hypothesized. Using advanced generations from the crosses, we further (c) asked whether the advantageous abiotic stress‐tolerance and herbivore‐defense traits segregated independently or nonindependently like an evolutionary trade‐off driven by common regulatory (pleiotropic) genes. This test was conducted in the laboratory under experimental drought‐stress and in the field across naturally occurring low elevation range boundaries. After implicating a pleiotropic‐driven trade‐off in this way, we used a targeted genetic linkage analysis to (d) determine whether a region on the *B. stricta* genome containing a candidate major effect TF was associated with the trade‐off. Because a genetic association between marker and trade‐off indicated evolutionary potential for overcoming the trade‐off, we also (e) measured selection on the traits across the range boundary for each marker genotype.

## 2 METHODS

### 2.1 Experimental organism

**Figure 1:**
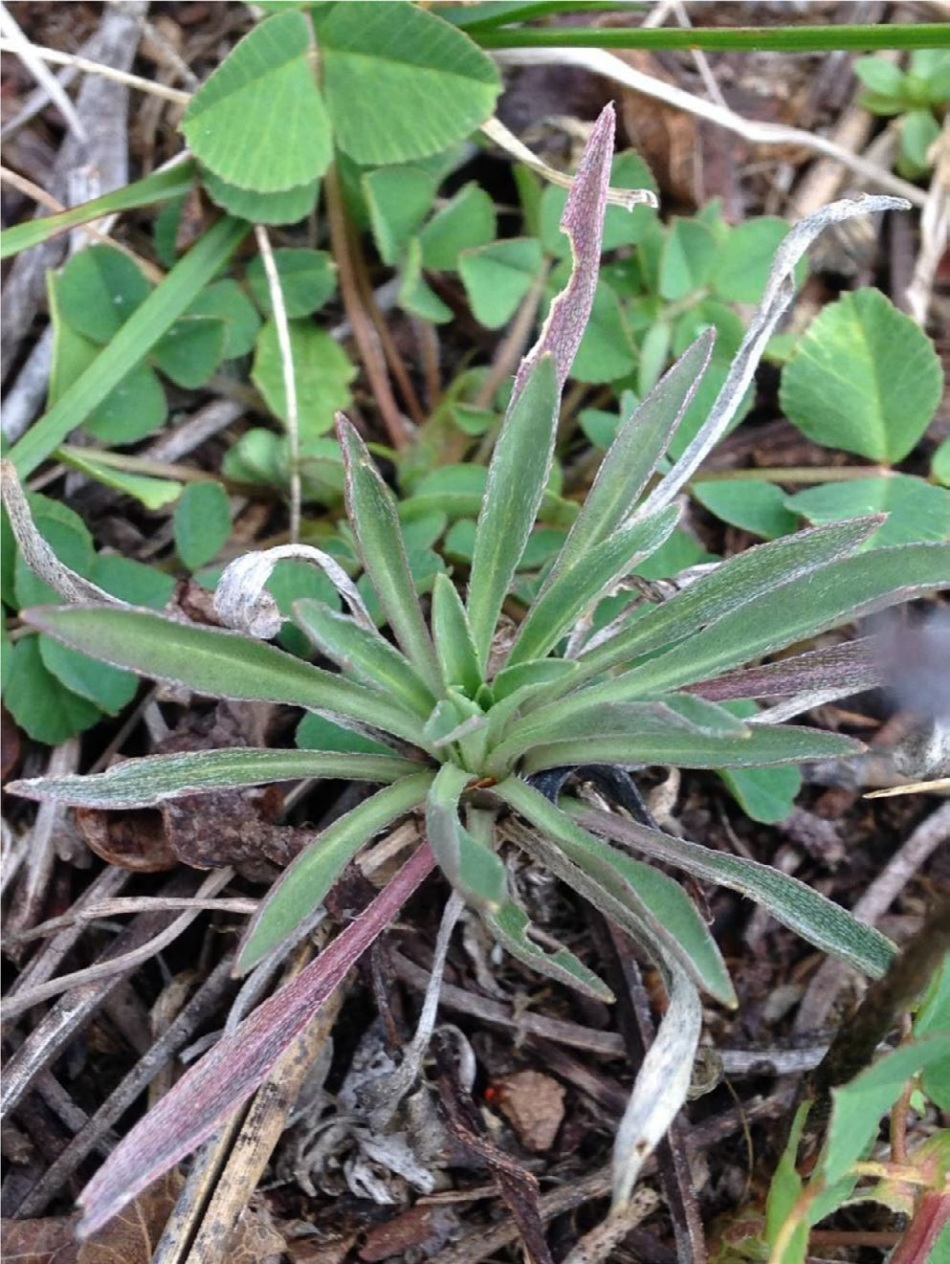
*Boechera stricta* basal rosette

*Boechera stricta* (Figure 1) is an emerging ecological model species that inhabits environments that differ substantially in drought‐stress, herbivore community, and other abiotic and biotic conditions throughout the Rocky Mountains and other upland regions of western North America (Rushworth et al., 2011). A close perennial relative of Arabidopsis, the genome of *B. stricta*, is also now sequenced and partially annotated (https://phytozome.jgi.doe.gov/pz/#!info?alias=Org_Bstricta). *Boechera stricta* predominantly self‐fertilizes (selfing rate in the northern portion of the range = 0.95, Song, Clauss, Pepper, & Mitchell‐Olds, 2006), enabling the creation of experimental advanced generation hybrids for ecological genetic studies (Prasad et al., 2012).

### 2.2 Populations

Extended generation crosses were conducted between plants of *B. stricta* from geographically and genetically isolated high and low elevation mountain ranges in the USA. Seed was collected from the Big Horn Mountains, WY (44°18′22″N, 107°18′33″W) at 2,780 m on the southeast side near Battle Park trailhead in sagebrush habitat and from the northern Black Hills, SD (44°24′50″N, 103°56′18″W) at 1,365 m, 11.25 km west of the town of Spearfish on Tinton road in Ponderosa Pine understory. These populations are close to the high and low elevation range limits of *B. stricta* in these mountain ranges and in general throughout much of the geographic range of this species (Song et al., 2006). The common garden experiments conducted across the low elevation range boundary were conducted in the same area where seed was originally collected in the Black Hills.

In the Black Hills, local range boundaries often coincided with low elevation range boundaries. As detailed elsewhere (Siemens, Haugen, Matzner, & VanAsma, 2009), just a few meters beyond these local range boundaries, conditions of lower elevations or climate change persisted, such as warming, water deficiencies, and increased herbivory, yet were otherwise similar to conditions within the range. Thus, by conducting experiments just across these local range boundaries, we minimized the number and effects of unmeasured correlated environmental factors. The two sites used for common garden experiments were 1 km apart representing separate local range boundaries.

### 2.3 Crosses

Using four inbred lines from each region (Tables S1 and S2), we conducted seven crosses between the high elevation Big Horn Mountains and low elevation Black Hills. We used the segregating F2 generation for analysis, and we used the next generation (F3) from two of the crosses (A and F, Table S1) in a field experiment across low elevation range boundaries.

### 2.4 Parent lines for reference and for measures of selection

#### 2.4.1 Growth chamber drought‐stress experiment

##### Experimental design

Representatives of the inbred lineages that were used as parents in crosses from each region were grown for reference to judge defense and stress‐tolerance characteristics in F2 plants. Four replicates of each of the eight parental lines (four from each region) were randomized into each of four flats for *N* = 128 parental line plants (2 populations/flat × 4 lines/pop × 4 reps/line × 4 flats = 128 plants from parent lines). Control and drought treatments were administered sequentially to every plant as described below under “F2 growth chamber experiment.” Timing and collection of drought tolerance and GS production data are also described in the “F2 growth chamber experiment” below.

##### Growth conditions

Seeds were planted in 0.2 L pots filled with a soil mix of 2/3 *Premier ProMix BX* and 1/3 sand. Pots also contained 46 mg of 7:40:6 NPK MagAmp time release fertilizer. Plants were grown in a BioChambers growth room with a 16/8 hr D/N photoperiod and 23/21°C D/N temperatures. Light intensity was 360 µmol m^−2^ s^−1^ from a combination of 1220 mm T5HO fluorescent and halogen lamps.

##### Statistical analysis

To determine whether there were genetic differences between high and low elevation parent populations in drought‐stress‐tolerance and chemical defense production in our experiment, we used the following general linear model in the GLM module of SYSTAT 13:

(1) $$\text{Response}=C+\text{Pop}+\text{Gtrt}+\text{flat}+\left(\text{Pop}\times \text{Gtrt}\right)+\text{development}$$

where the response variable (Response) was either the GS production or the drought‐stress‐tolerance response, C was a constant, Pop was for the two populations, Gtrt was a factor to help control for any inherited environmental effects (see Table S2 for further explanation), and development was seedling size to control for any correlated developmental differences. Because both GS and tolerance variables were multivariate (see below under “Defense” and “Drought tolerance”), each of the analyses was necessarily MANCOVAs. When factors were significant in the MANCOVA, subsequent univariate tests were protected from Type I errors that would otherwise occur from multiple testing of each response variable separately (Montgomery, 1997). However, in the case of the GS production variables, correlations among the GS production variables did not allow for MANOVA in SYSTAT; therefore, we corrected for multiple testing following the ANCOVAs using the Bonferroni method (Rice, 1989). Interaction terms involving flat and development (seedling size) were eliminated from the model to simplify after determining that these interactions were not significant and did not affect factors of interest. Therefore, flat was a blocking factor to control for any unmeasured environmental effects among flats, and seedling size was a covariate to control for development. Because the replicates from each population were randomized together within each flat, any differences among populations were attributed to genetically diverged differences.

#### 2.4.2 Field experiment across low elevation range boundary

##### Experimental design

The parental inbred lines from each region were also grown for reference in the F3 field experiment (see description of Section 2.5.2 below) and to examine selection on tolerance and resistance traits. Ten replicates from each of four parent lines (the parent lines of crosses A and F) were randomized into blocks at the two, low elevation range boundary sites (Siemens et al., 2009 for boundary description). At each site, one block was placed within the range, and one block was placed just outside the range about 15 m away. There were 160 plants total in this parent line field experiment (10 replicates/line x 4 lines/block × 2 blocks/site × 2 sites = 160 plants). Plants were started in plug flats in a growth chamber and then transplanted to the field 2 weeks later. In the field, plants were separated within blocks by 10 cm; therefore, just as in nature, nearest neighbors were other species of forbs in these diverse meadow habitats (Siemens & Haugen, 2013).

##### Response variables

We used the extent of leaf color change on a 0–5 visual scale as an indicator of abiotic stress for plants that had not yet been fed upon by herbivores. Under abiotic stress, such as drought, *B. stricta* basal rosette leaves turn a red violet color (Gill, Haugen, Matzner, et al., 2016), most likely because of Betacyanin production. Betacyanins are produced in response to abiotic stress such as drought and function as ROS scavengers (Casique‐Arroyo, Martínez‐Gallardo, González de la Vara, & Délano‐Frier, 2014). Some of the random unmeasured variation inherent in such a subjective scoring was controlled for statistically in the blocking factor because the same individual researchers scored all the plants within blocks and the same number of blocks within and outside the range. We did not attempt to use the color change in any measure of tolerance to abiotic stress.

To determine the effects of range boundary on abiotic stress‐tolerance, we analyzed fitness‐correlated growth rates (see Section 3). The width of basal rosettes was measured using digital calipers, just before transplanting, and then in the field 14 and 24 days post‐transplanting in October. Tolerance was quantified as how well the plants grew outside the range relative to growth within the range, that is, the difference, controlling for size at transplanting by subtracting size at transplanting from each measurement, and by using plant size as a covariate in the analysis (see below under Statistical analysis). For each population, tolerance is defined as a reaction norm of performance under stress relative to control conditions (Simms, 2000). The range boundary‐by‐population interaction indicates differences between the populations in stress‐tolerance, in the ANCOVA—analysis of covariation (SYSTAT 13.1).

Each time plant size was measured, area consumed by herbivores was measured with a clear grid of 1 mm^2^ squares laid over the plant. Damage by specialists was readily identifiable “buckshot hole” patterns by flea‐beetles (genus unknown). All other chewing damage was assumed to be done by generalists.

##### Statistical analysis

To determine the effects of the low elevation range boundary on plants from the high and low elevation populations, we conducted logistic regression or ANCOVA in SYSTAT 13, depending on the response variable of interest. For analysis of overwinter survivorship and frequency of attack by generalist herbivores, we used logistic regression because the response variable in each case was binary; dead or alive; and attacked or not, respectively. The factors that were included in these logistic regressions included range boundary, population, and the interaction between boundary and population.

To determine the effects of the range boundary on an indicator of abiotic stress, tolerance to abiotic stress, and to determine whether there were associations between relevant traits and survivorship, we conducted ANCOVA in GLM of SYSTAT 13. In each case, the basic statistical model used was:

(2) $$\text{Response}=C+\text{Pop}+\text{Range}+\left(\text{Pop}\;\times \;\text{Range}\right)+\text{site}+\text{development}$$

where C was a constant, Pop represented the two populations (high and low elevation), Range was the low elevation range boundary (within and just outside), site was the two field sites containing low elevation range boundaries, and development was seedling size at the time of transplantation. Interactions involving site or development and other factors were dropped from the analysis to simplify because they were not significant (*p*'s >> .05) and did not change the detection of effects of interest.

To determine whether there was selection for increased tolerance and resistance across the low elevation range boundary, we analyzed for associations between these traits and fitness (overwinter survivorship). We did this by adding survivorship to the statistical model #2, including interaction terms with population and range.

### 2.5 Do defense and stress‐tolerance traits segregate independently?

#### 2.5.1 F2 growth chamber experiment

##### Experimental design

For each of seven crosses between the high and low elevation populations, 128 F2 sibs were planted across four blocks (planting flats) for a total sample size of 896 plants (7 crosses × 4 planting flats/cross × 32 pots/flat = 896 F2 plants). All flats were rotated 180° and moved to a different location within the chamber every other day to minimize unmeasured random environmental variance.

##### Drought treatments

Control and drought treatments were administered sequentially to every plant, rather than having separate sets of control‐ and drought‐treated plants (Figure S1). Ideally, stress‐tolerance is measured as performance of a replicated genotype across a stress gradient (Simms, 2000). However, in the F2 segregating populations, there was no identifiable replication of genotypes. Instead, performance (i.e., growth) for each F2 plant was monitored during control and then drought‐stress periods for a drought‐stress‐tolerance measure for each plant.

Gradual drought conditions were imposed until plants were observed having reversible wilting, and then maintained just above these levels through the drought treatments, as done previously (Alsdurf, Anderson, & Siemens, 2015; Alsdurf, Ripley, Matzner, & Siemens, 2013). Flat weights were measured on postal scales to monitor watering (Figures S1 and S2). From previous experiments (Alsdurf et al., 2013), we knew the approximate flat weights needed to impose drought‐stress such that growth is reduced relative to controls but remains positive.

##### Drought tolerance

For estimates of drought tolerance, we measured relative growth, leaf mass area (LMA), and root:shoot ratio. Several measures of stress‐tolerance increase the probability of detection and provide a general assessment (Donovan, Maherali, Caruso, Huber, & Kroon, 2011). Relative growth rates were used to assess drought tolerance because in the process of decreased turgor and subsequent ABA synthesis caused by water deficiencies, growth of shoots decreases before photosynthesis and subsequent wilting (Fitter & Hay, 2002). Differences in rosette size during week‐long growth periods, in each of control and drought periods, were used to measure growth rates (Figure S1).

Tolerance compensates for losses, and LMA and root:shoot ratio are compensatory responses to unsustainable water loss by decreasing water loss and/or increasing water uptake, respectively. Four leaves collected from each plant were freeze‐dried for GS analysis (see below under “Defense”) and were used to estimate LMA. LMA for each plant was calculated as the average weight/width of these four leaves. For root:shoot ratios, fresh and freeze‐dried weights were obtained for whole shoots (basal rosettes) and roots. Roots were floated in water and rinsed to remove sand and soil materials before weighing and freeze drying.

##### Defense

On day 36 postplanting (Figure S1), four leaves from the middle whorl of each rosette were collected for GS analysis. Leaves were immediately flash frozen in liquid nitrogen and then freeze‐dried. Glucosinolates were extracted from the leaves in methanol, isolated on Sephadex ion‐exchange columns, and measured on a HPLC (Brown, Tokuhisa, Reichelt, & Gershenzon, 2003; Prestera et al., 1996) as summarized elsewhere (Alsdurf et al., 2013). Briefly, weighed, freeze‐dried basal rosette leaves were extracted in 1.2 ml methanol, separated on a 0.6‐ml DEAE A‐25 Sephadex column, and eluted after 12 hr incubation with sulfatase (Sigma‐Aldrich). A Lichroshpere (RP‐C18, endcapped) 250 × 4‐mm analytical column was used on the HPLC, and chromatograms generated at 229 nm were analyzed.

##### Statistical analysis

To determine whether there was a negative genetic association between defense production and drought‐stress‐tolerance in the F2 segregating populations, we controlled for variation among crosses, any unmeasured random variation among planting flats, and any developmental differences. Developmental differences among plants were controlled by including initial seedling size measures in the statistical model as covariates. Because there were four flats for each cross, flat was nested within cross. The statistical model used was:

(3) $$\text{Tolerance}=C+\text{Cross}+\text{Defense}+\left(\text{Cross}\;\times \;\text{Defense}\right)+\text{flat}\left(\text{Cross}\right)+\text{development}.$$

where C is a constant. Because there were several measures of tolerance and defense, we used multivariate analysis of variance in SYSTAT 13 for analysis. We included the three measures of drought tolerance (root:shoot ratio, LMA, and relative growth) as dependent variables, and then either total or individual GS concentrations, or the ratio of BCGS/MET‐GS concentrations as the independent Defense variable. Interaction terms involving “flat(Cross)” or “development” were eliminated from the analysis to simplify after determining that these effects were not significant (*p*‐values » .05) and did not detract from other effects of interest.

#### 2.5.2 F3 field experiment

##### Experimental design

The F3 generation allowed us to test for the trade‐off across the range boundary. In self‐fertilizing species like *B. stricta*, an F3 family essentially represents replication of each parent F2 genotype. Thus, with replication of each genotype, we were able to split each F3 family in the field experiments, half planted within the range, and half outside the range. Further, full‐sib family mean phenotypic values in the F3 generation were mapped using F2 genotypic information because *B. stricta* is self‐fertilizing (Schranz, Dobe, Koch,& Mitchell‐Olds 2005).

The design of the field F3 experiments was split plot. The experiments, for each cross, were set up at two different sites in the Black Hills representing local low elevation range boundaries. For each cross at each site, two replicates of each of 20 F3 families were randomized into each of six blocks (2 crosses × 2 sites × 2 plants/family × 20 families × 6 blocks/cross/site = 960 plants). Three of the blocks were within the local range, and three were just 15 m away outside the range. Replicated blocks were 5 m away from one another, and as in the Parental line experiment, plants of *B. stricta* within blocks were at 10 cm centers. In each cross, we used families (seed) from twenty extreme F2 parents: ten of the F2 parents were high in GS concentrations, but low in a measure of drought tolerance, while the opposite for the other ten. In mid‐September, blocks in the F3 experiments were started in plug flats in the growth chamber, and then the seedlings were transplanted 2 weeks later.

##### Response variables

Plants in the field experiment were monitored for 1 year; plants were monitored for growth and herbivory in the fall and then for overwinter survivorship in the spring. In the fall, survivorship did not change appreciably from the spring census. Two weeks after transplanting, we began measuring plant size, area consumed by herbivores, and the Betacyanin leaf color score, as in the parent line experiment.

Tissue of the F3 families was used to measure carbon isotope ratios for another assessment of stress and stress‐tolerance. Water use efficiency (WUE) is the ratio of CO_2_ uptake to water loss. The carbon isotope ratio (δ^13^C) is also used to estimate WUE in C_3_ plants (Farquhar & Richards, 1984; McKay, Richards, & Mitchell‐Olds, 2003 and references therein). The δ^13^C works as a surrogate for WUE because the ^13^C/^12^C ratio can be modeled as a function of the ratio of intercellular to atmospheric partial pressure of CO_2_ (C_i_/C_a_), and C_i_/C_a_ is empirically correlated with WUE in C3 plants. Less negative values of δ^13^C indicate greater WUE. For carbon isotope discrimination, whole basal rosette shoots were freeze‐dried, ground to <0.5 mm, and analyzed on a Thermo Delta V isotope ratio mass spectrometer (IRMS) interfaced to a NC2500 elemental analyzer at the Cornell Isotope Laboratory (COIL). Values were expressed as per ml (‰) ^13^C values.

##### Statistical analysis

To determine whether defense and abiotic stress‐tolerance across the range boundary segregated independently from one another, we conducted a parent–offspring regression (Conner & Hartl, 2004) for each cross. Abiotic stress‐tolerance across the range boundary was from F3 families, and GS values from F2 parents. We used F3 family mean values after controlling for random environmental variation among plots and for development. Similarly, we used F2 parent values after controlling for random environmental variation among flats and for development. Thus, for each cross, the regression model was:

(4) $$\text{Tolerance}=C+\text{site}+\text{GS}+\left(\text{site}\;\times \;\text{GS}\right)$$

where Tolerance was abiotic stress‐tolerance, C was a constant, site was variation between the two field sites, GS was F2 parent GS ratio value, and (site × GS) was the interaction term. Tolerance was relative growth from plants that had not yet been damaged, as explained above for the parent lines. We used GS ratio because of its relevance to resistance to generalists herbivores in the field (Prasad et al., 2012) and because it stood out in the marker association analysis (see Section 3).

### 2.6 Genotyping

#### 2.6.1 Markers linked to candidate genes

Of primary interest was the microsatellite marker R6_B06 that was located at the site of TF AtMYC2 [At1g32640] on the *B. stricta* genome. Chromosomal painting and end sequencing has shown that there are large syntenic blocks that align between the *Arabidopsis thaliana* and *B. stricta* genomes (Schranz, Windsor, Song, Lawton‐Rauh, & Mitchell‐Olds, 2007). The microsatellite R6_B06 was amplified by PCR as in Song et al. (2006) and genotypes elucidated by electrophoresis on a metaphor gel. DNA was extracted using DNeasy Plant Mini Kit (Qiagen) according to manufacturer's protocol from all F2 plants of crosses A and F (Table S1).

##### Statistical analysis

We determined whether there was an association between genetic variation in the trade‐off and genotypic variation in marker R6_B06, which was linked, in the crosses, to the candidate TF MYC2. This was done for two of the crosses, A and F, in the F2 and F3 generations. In the F2 generation, we examined the effects of R6_B06 genotypic variation on the genetic correlation between glucosinolate production and stress‐tolerance. Because there were several potential GS production variables that mattered, this was done by first constructing principal components (PCs) on *n* = 8 GS production variables (Total GS, BCGS1, BCGS2, METGS, BCGS/METGS, BCGS1/METGS, BCGS2/METGS, BCGS1/BCGS2). PCs that explained at least 1/*n* = 1/8 or 12.5% of the variation were considered significant (Afifi & Clark, 1984) and used in subsequent analyses. We also reduced the dimensionality of the three stress‐tolerance variables (root:shoot ratio, RELG, LMA) for subsequent analysis by first standardizing each variable and then adding the three standardized variables together for one stress‐tolerance axis. For each of the crosses A and F in the F2 experiment, we then used the following GLM to examine the effect of R6_B06 genotypic variation on the relationship between the multivariate GS production and stress‐tolerance variables:

(5) $$\text{GSPCs}=C+\text{R6\textbackslash{}\_B06}+\text{tolerance}+\left(\text{R6\textbackslash{}\_B06}\;\times \;\text{tolerance}\right)+\text{flat}+\text{development}$$

where GSPCs represented the significant GS production PCs; C was a constant; R6_B06 was the three marker genotypes (two homozygotes and the heterozygote); tolerance was the summed standardized stress‐tolerance variables; Flat was planting flat, a blocking factor; and development was freeze‐dried weight of the leaves used for GS analysis, which was correlated with plant size (*r* = .89). We were particularly interested in the interaction term in the model, which indicated that the correlation between GS production and stress‐tolerance was dependent on genotypic variation of the marker. The analysis was multivariate because we used the first three PCs in the analysis, as each explained 51.7%, 31.1%, and 16.9% of the total variance in the GS production variables.

In the association analysis of the F3 field experiment, we were interested in whether natural selection on GS production and stress‐tolerance traits across the range boundary changed depending on the genotype of the marker R6_B06. The analysis in the case of GS production was a parent–offspring regression, using the GS production values from the F2 parent and the mean F3 offspring family value of survivorship. In this case, the GLM model used was.

(6) $$\text{Survivorship}=C+\text{BCGS}/\text{METGS}+\text{R6\textbackslash{}\_B06}+\left(\text{BCGS/METGS}\;\times \;\text{R6\textbackslash{}\_B06}\right)$$

Here, Survivorship was the proportion of the survivors in each F3 family; C was a constant; BCGS/METGS was the ratio of branch‐chain to straight‐chain—methionine derived—glucosinolates; and R6_B06 was genotypic variation in the marker.

For stress‐tolerance, we used the F3 family mean value calculated from relative growth rates across the low elevation range boundary. The GLM model was.

(7) $$\text{Survivorship}=C+\text{Tolerance}+\text{R6\textbackslash{}\_B06}+\left(\text{Tolerance}\;\times \;\text{R6\textbackslash{}\_B06}\right)$$

In the models 4 and 5, we were particularly interested in the interaction terms, indicating that selection on the trait of interest changed according to the genotype of the marker.

## 3 RESULTS

### 3.1 Parent lines for reference and for measures of selection

#### 3.1.1 Growth chamber drought‐stress experiment

The parental lines from the low elevation Black Hills population had higher concentrations of each of the three common GS (GS): two branched‐chain GS (BCGS), 2‐hydroxyl‐1‐methylethyl (BC‐GS1), and 1‐methylethyl (BC‐GS2), plus the straight‐chain GS 6‐methylsulfinylhexyl (MET‐GS). The populations also differed significantly in total GS content and the ratio of BCGS/MET‐GS (Table 1 and Table S3 for statistical analysis). However, this difference between populations was mainly caused by higher levels of BCGS in the Black Hills.

**Table 1:** Documentation of stress‐tolerance and herbivore‐defense trait differences between the high (Big Horn) and low (Black Hills) elevation populations represented by the parental lines used in the crosses

	Stress‐tolerance				Glucosinolate production				
	LMA*	RELG***	Wet‐ R:S***	Dry‐ R:S**	Total‐ GS***	BC‐GS1***	BC‐GS2***	MET‐GS*	GS‐ ratio***
Big Horns	0.046 (0.002)	1.272^a^ (0.128)	3.735 (0.168)	2.324 (0.142)	601.948 (86.528)	439.703 (57.834)	117.077 (18.744)	61.864 (6.534)	9.381 (0.701)
Black Hills	0.051 (0.001)	0.456 (0.109)	2.730 (0.148)	1.877 (0.130)	1,105.526 (75.681)	765.101 (51.751)	184.396 (16.707)	79.466 (5.585)	12.624 (0.599)

Note

Least squares means (+1*SE* in parentheses) of drought‐stress‐tolerance and glucosinolate production variables are shown. Statistical analyses in Tables S3 and S4. Stress‐tolerance variables and abbreviations: leaf mass area (LMA), relative growth rate (RELG), wet root:shoot ratio (Wet R:S), dry root:shoot ratio (Dry R:S). Glucosinolate production variables and abbreviations: BCGS1 is 2‐hydroxyl‐1‐methylethyl GS, BCGS2 is 1‐methylethyl GS, METGS is 6‐methylsulfinylhexyl GS, GS Ratio is (BCGS1 + BCGS2)/METGS. See Tables S3 and S4 for statistical analysis. Units for LMA is mg/mm, and for GS, µmol/mg. RELG, R:S and GS ratio do not have units because each is a ratio of the same units (growth rates, weights, and concentrations, respectively).

a Values are −log‐transformed.

* *p* ≤ .05,

** *p* ≤ .01,

*** *p* ≤ .001.

In contrast to the lines from the low elevation population, the lines from the high elevation Big Horn population had a greater root:shoot mass ratio and a greater decrease in relative shoot growth rate (−log(RELG)) (Table 1, Table S4 for statistical analysis). We detected no great difference in LMA between the populations, yet still significant, despite slightly higher levels in the Black Hills population.

#### 3.1.2 Field experiment across low elevation range boundary

**Figure 2:**
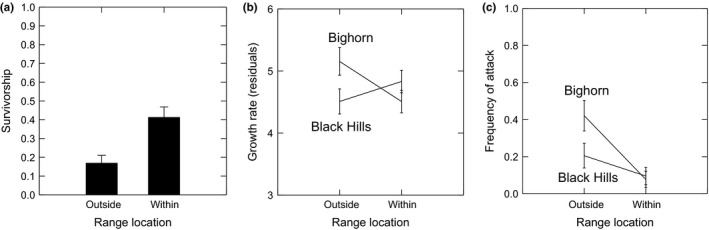
Effect of the low elevation range boundary on *Boechera stricta* (a) overwinter survivorship (Logistic regression of the effect of range boundary: *Z* = 2.153, *p* = .031), and (b) growth rates (Range‐by‐population interaction in ANCOVA Table S6: *F*_1,114_ = 7.630, *p* = .007) and (c) frequency of attack by generalist insect herbivores (Range‐by‐population interaction in Logistic Regression: *Z* = −3.893, *p* < .001) that contributed to lower survivorship across the range (Figure S4). Error bars are ±1*SE*. Residuals are least squares means after controlling for site and development. Lower survivorship across the range boundary indicates that evolution by natural selection is a likely response needed for range expansion. Greater growth rates outside the range relative to within the range indicate greater tolerance to the stress experienced outside the range. Growth rates were calculated from plants that had not yet been damaged by herbivores; therefore, it is assumed that the tolerance was to abiotic stress. Also see Figure S3 and Table S5 for Betacyannin color score results, another correlated indicator of abiotic stress across the low elevation range boundary. Note that attack rates by generalist herbivores also increase outside the low elevation range boundary

Plants of both high and low elevation populations had lower fitness (overwinter survivorship) across the low elevation range boundary (Logistic regression: *Z* = 2.153, *p* = .031; Figure 2a). There was no difference between the populations in this effect of the range boundary (no Range boundary‐by‐Population interaction in the logistic regression: *Z* = 0.330, *p* = .741). And there was no difference between the populations in survivorship (i.e., no main effect of Population in the logistic regression: *Z* = −0.602, *p* = .547).

Coincident with low survivorship across the low elevation range boundary was an increase in the violet‐red color of leaves (*F*_1,112_ = 25.435, *p* < .001, Figure S3 and Table S5), indicative of a Betacyanin response to abiotic stress. This analysis was conducted on plants that had not yet been attacked by herbivores; therefore, this response was likely due to abiotic stress. The plants had not yet been attacked because measurements were made early in the experiment not because of a resistant subset of plants. Later in the fall, many more plants of both populations were fed upon. There was also no difference between populations in the effect of the range boundary on this indicator of abiotic stress (no Range boundary‐by‐Population interaction: *F*_1,112_ = 0.940, *p* = .334). However, a consistent difference between populations within and outside the range was detected (*F*_1,112_ = 6.646, *p* = .011); the low elevation Black Hills population had the higher color scores.

Although the area across the low elevation range boundary was a more stressful environment to plants of both populations, demonstrated by lower survivorship and higher Betacyanin color scores, plants from the high elevation Big Horn population had higher tolerance to the abiotic stress measured as growth rates outside the range relative to inside the range (Range‐by‐population interaction: *F*_1,114_ = 7.630, *F* = 0.007, Figure 2b and Table S6). This tolerance can be attributed to the abiotic stress as the growth rates were taken from plants that had not yet been attacked by herbivores. This difference between populations in abiotic stress‐tolerance outside the low elevation range boundary was predicted based on differences in performance (i.e., root:shoot mass ratio) in the drought‐stress growth chamber experiment (Table 1).

The frequency of attack by generalist insect herbivores was also greater in the area outside the low elevation range boundary for both populations, but plants from the high elevation Big Horn population were disproportionately attacked more frequently outside the range (Range‐by‐population interaction in Logistic Regression: *Z* = −3.893, *p* < .001; Figure 2c). This result was also predicted based on the differences between populations in GS production (Table 1).

Lower stress‐tolerance and greater damage by herbivores were both associated with lower fitness (overwinter survivorship) across the low elevation range boundary. Growth rate (used to calculate tolerance) was associated with survivorship (*F*_1,111_ = 9.182, *p* = .003, Table S7). The association between growth rate and survivorship was conducted on plants that had not been fed upon by herbivores in the fall census. Consequently, the overwinter mortality was caused mainly by abiotic stressors. Further, the association between growth and survivorship was dependent on population and range boundary (Table S7). The marginally significant (*p* < .1) three‐way interaction (Population‐by‐Boundary‐by‐Survivorship interaction; *F*_1,111_ = 3.376, *p* = .069; Figure S4a) suggests that mortality was associated with lower relative growth (i.e., lower abiotic stress‐tolerance) just across the low elevation range boundary for plants from the low elevation Black Hills population. Mortality of Big Horn plants across the range boundary could not be attributed to low growth rates.

Damage by herbivores was also associated with lower survivorship (*F*_1,141_ = 6.616, *p* = .011, Table S8). This association between herbivory and survivorship was dependent on range location (*F*_1,141_ = 7.103, *p* = .009, Table S8 and Figure S4b). Plants that did not survive overwinter outside the range had been fed upon more in the fall. We did not find that the association between survivorship and herbivory differed between populations (i.e., no significant three‐way interaction; Table S8).

### 3.2 Do defense and stress‐tolerance traits segregate independently?

#### 3.2.1 F2 growth chamber experiment

GS production and drought‐stress‐tolerance did not segregate independently from one another in the F2 mapping populations (Multivariate analysis: Table 2). This was mainly true for drought‐stress‐tolerance measured as relative growth (−log(RELG)) or leaf mass area (LMA) (Univariate analysis: Tables S9 and S10). However, RELG differed between high and low elevation populations (Table 1) and is informative for linkage mapping in the F2 and F3 segregating generations.

**Table 2:** Statistical analyses that determined whether glucosinolate (GS) defense and drought‐stress‐tolerance variables were independent of one another in an advanced generation of the crosses between high and low elevation populations

Source	*df*	Total GS	BCGS1	BCGS2	METGS	GS Ratio
GS	3, 502	20.624***	14.505***	39.611***	18.639***	5.164**
Cross	15, 44	1.276	0.780	1.356	1.737	0.524
GS x Cross	15, 1,386	3.871***	3.894***	3.107***	4.438***	1.248
Flat	54, 1,496	6.191***	6.401***	6.203***	6.221***	5.716***
Seedling size	6, 1,004	12.030***	14.693***	6.073***	17.101***	18.173***

Note

Shown are F2‐generation F‐ratios from MANCOVAs on stress‐tolerance traits (R:S, RELG, LMA) as a function of GS production variables (Total GS, BC‐GS1, BC‐GS2, MET‐GS, GS ratio—see Table 1 for explanation of abbreviations).

* *p* ≤ .05,

** *p* ≤ .01,

*** *p* ≤ .001.

**Figure 3:**
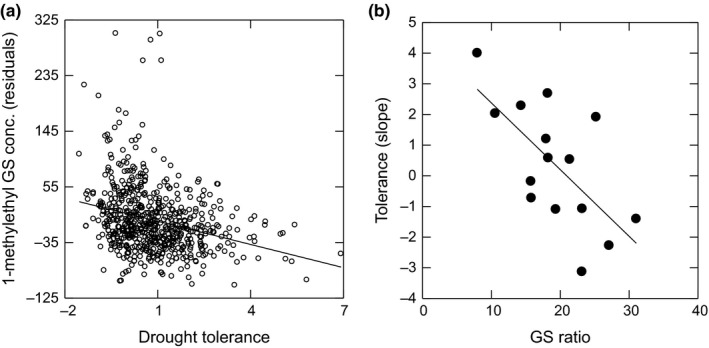
Trade‐off in the (a) F2 segregating population between 1‐methylethyl GS content and laboratory drought‐stress‐tolerance (*F*_1,599_ = 65.987, *p* < .001), and in the (b) F3 generation between GS ratio (branch chain/straight chain) and stress‐tolerance measured across the low elevation range (parent‐offspring regression: *F*_1,13_ = 10.333, *p* = .007, *r* ^2^ = 44.4%). Drought tolerance in the F2 experiment was −log(RELG), the characteristic growth of the abiotic stress tolerant high elevation Big Horn plants in the drought‐stress laboratory experiment (Table 1). Data in (a) are genetic variation among F2 individuals from seven crosses after controlling for random unmeasured variation among flats and plant development (size), hence the residuals. Data in (b) are genetic variation among F3 families from cross “A” at one site

There was a negative relationship between GS production and the measures of drought‐stress‐tolerance (e.g., 1‐methylethyl GS [BCGS1] vs. relative growth: Figure 3a). Only minor deviations from this trend were detected (e.g., GS‐by‐Cross interaction, Table 2, Figure S5). Thus, we detected a negative genetic correlation between GS production and stress‐tolerance, which is indicative of an evolutionary trade‐off.

In another analysis, the genetic correlation in the F2 population between GS production and stress‐tolerance varied significantly with genotypic variation at the R6_B06 marker (e.g., cross A, marker genotype‐by‐stress‐tolerance interaction in an ANCOVA on GS production: *F*_6,150_ = 2.653, *p* = .018). R6_B06 was linked to the candidate TF MYC2 in the crosses. This was a multivariate analysis using three significant GS PCs (principal components) together as response variables. In subsequent protected univariate analysis on each PC separately, PC1 and PC3 were significant (*p*'s ≤ .05). The highest component loading (0.95) on PC1 was for the BCGS/METGS ratio, while any one of the loadings did not stand out (loadings < 0.6) for PC3. This multivariate association analysis implicates the genome region containing MYC2 TF as a possible area of candidate genes for both the cause of the trade‐off and for evolutionary potential of the trade‐off.

#### 3.2.2 F3 field experiment

We also observed the trade‐off between GS production and range boundary stress‐tolerance in the field experiment across the low elevation range boundary (Figure 3b). Tolerance in the F3 population, measured as differential growth across the low elevation range boundary (i.e., the difference in growth rates between outside and within the range), was negatively genetically correlated with GS production in the F2 parents (parent–offspring regression: *F*_1,13_ = 10.333, *p* = .007, *r* ^2^ = 44.4%). However, for both crosses, the trade‐off was only observed at one of the sites (GS‐by‐Site interaction in the ANOVA on tolerance, Cross A—*F*_1,31_ = 11.312, *p* = .004, and Cross F—*F*_1,33_ = 4.885, *p* = .034). Here, the measure of GS production was the ratio of branch‐chain to straight‐chain GS, which is correlated with resistance to generalist insect herbivores (Prasad et al., 2012). Attack by generalist insect herbivores increases across the low elevation range boundaries (e.g., Figure 2c, see also Siemens et al. (2009)). We also used the GS ratio in the analysis because it stood out in the marker association analysis (see above). Similarly, when carbon isotope ratios were used as the measure of stress‐tolerance, the trade‐off was observed outside the range, but not within the range (Boundary‐by‐GS ratio interaction: *F*_1,25_ = 2.988, *p* = .096, Figure S6).

**Figure 4:**
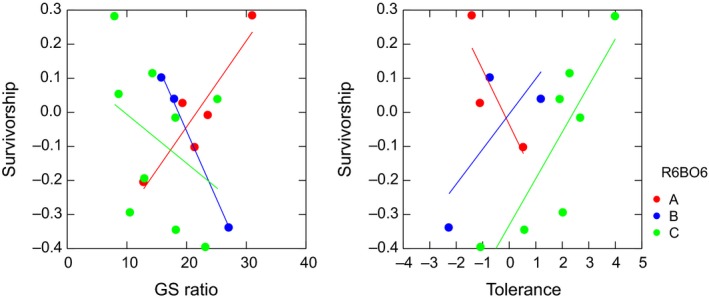
Evidence that evolution of the trade‐off is also constrained; selection gradients for the R6_B06 genotypes outside the range in the F3 common garden field experiment. For each marker genotype (A and B are the homozygotes, C the heterozygote), F3 families that had highest overwinter survivorship were either high in stress‐tolerance or GS ratio, but not both. Thus, although the selection gradients varied among R6_B06 genotypes for GS ratio (genotype‐by‐GS ratio interaction: *F*_2,11_ = 3.824, *p* = .055) and for tolerance (*F*_2,7_ = 3.238, *p* = .101), within each genotype, the gradients for GS ratio and tolerance had opposite signs

Despite evidence for evolutionary potential in the trade‐off (i.e., significant association with R6_B06 marker variation), the selection gradient for each genotype changed such that there was never selection for high values of both traits. Within each R6_B06 genotype, F3 families that had the highest fitness across the range were either high in GS ratio or tolerance, but never both (Figure 4). That is, for each trait, the selection gradient changed (marginally significant—*p*'s ≤ .1) depending on R6_B06 genotype (marker genotype‐by‐GS ratio interaction: *F*_2,11_ = 3.824, *p* = .055, and genotype‐by‐tolerance interaction: *F*_2,7_ = 3.238, *p* = .101), but there was never simultaneous selection for high values of both traits.

## 4 DISCUSSION

Several factors have been proposed that might constrain adaptation at range boundaries (Sexton et al., 2009). Seed dispersal potential and soil germination experiments indicate that barriers to dispersal or establishment cannot explain local range boundaries of *B. stricta* (Siemens et al., 2012). Gene flow or lack of genetic variation is also unlikely constraint because population genetic studies indicate great differentiation throughout the geographic range of *B. stricta* (*F*_ST_ = 0.56), half of which segregates within populations (Song et al., 2006, 2009). This population genetic differentiation and variation also include the high and low elevation range margin populations studied here (Siemens & Haugen, 2013). There is also significant quantitative genetic variation for the relevant traits (resistance to herbivores, GS content, abiotic stress‐tolerance) in low elevation range margin populations of *B. stricta* (Siemens et al., 2009; Siemens, Keck, & Ziegenbein, 2010).

Instead, family‐structured quantitative genetic studies have provided correlative evidence for the existence of an evolutionary trade‐off between defense and abiotic stress‐tolerance in *B. stricta* (Siemens et al., 2009). Such trade‐offs may be caused by linkage disequilibrium or pleiotropy (Conner & Hartl, 2004). To experimentally test for the trade‐off and its molecular basis, we crossed high and low elevation populations that have diverged for defense and stress‐tolerance quantitative traits (Anderson, Perera, Chowdhury, & Mitchell‐Olds, 2015 for other populations like these).

Common garden field experiments comparing the populations showed that high defense levels of the low elevation Black Hills population and the high abiotic stress‐tolerance levels of the high elevation Big Horn population were both favored by natural selection across the low elevation range boundary. Similar results have been found for quantitative genetic variation within range margin populations of *B. stricta* (Siemens & Haugen, 2013; Siemens et al., 2009). The simultaneous evolution of defense and abiotic stress‐tolerance traits as the range expands into lower elevations, or for adaptation to climate change as low elevation range boundaries shift upslope, should be predicted.

However, the defense and stress‐tolerance traits did not segregate independently from one another in the F2 or F3 mapping populations in either the laboratory or field, respectively. This experimental genetic result supports previous correlative genetic studies (Alsdurf et al., 2013; Siemens et al., 2012, 2009; Siemens & Haugen, 2013). Thus, the trade‐off could contribute to range limit development by acting as an evolutionary constraint preventing the simultaneous evolution of defense and stress‐tolerance needed for range expansion.

The marker association analysis further provided a molecular test of pleiotropy and allowed us to evaluate a region on the genome containing a candidate gene. Candidate pleiotropic genes for the trade‐off included TFs, which can coregulate ecological responses to simultaneous challenges of abiotic and biotic stressors (Atkinson & Urwin, 2012). In apparent agreement with this prediction, genetic variation in the trade‐off was associated with genotypic variation in marker R6_B06, which was linked in the crosses to candidate TF MYC2. However, significant associations in linkage analysis implicate gene regions, not individual genes. Further, pairwise sequence comparison of resequenced MYC2 coding regions (Olsen[Link], unpublished data) and of differentially expressed MYC2 transcripts sourced from RNASeq data (Gill, Haugen, Matzner, et al., 2016), however, have revealed no polymorphisms between the high and low elevation populations studied here (Olsen[Link], unpublished data). We hypothesize, instead, that if MYC2 is involved in the trade‐off, there may be sequence variation in *cis* regulatory regions of MYC2.

Nonetheless, we argue that there must be genetic variation in a regulatory gene for the significant association between marker and trade‐off to occur. Interestingly, this covariation between molecular marker and trade‐off could also be interpreted as evolutionary potential to overcome the trade‐off. However, we did not find that this variation would break the trade‐off. Within each marker R6_B06 genotype, F3 families that had the highest fitness across the range boundary were either high in GS ratio or tolerance, but never both. This analysis indicated that natural selection on regulatory genes linked to the marker may not change the negative genetic correlation between GS production and stress‐tolerance, possibly because of additional pleiotropic effects on other unmeasured traits.

For evolutionary inference, evolutionary ecologists have defined and detected trade‐offs as negative genetic correlations among traits that affect fitness (Conner & Hartl, 2004), and more recently using linkage or association mapping for molecular evidence of pleiotropy (Anderson, Lee, Rushworth, Colautti, & Mitchell‐Olds, 2013). Experimental studies regarding range limits have given mixed results regarding the possible role of trade‐offs. Some show weak correlations between genetics and traits suggesting low constraint on range edge evolution (Gould et al., 2014) while others show significant constraints (Colautti & Barrett, 2013; Etterson & Shaw, 2001). In general, more traditional life history traits that may be involved in trade‐offs may not be good predictors of range dynamics (Comte, Murienne, & Grenouillet, 2014). To improve the study of defense trade‐offs here, we (a) measured the well‐defined GS defense physiology, used (b) growth characteristics of the high elevation population as a reference to judge abiotic stress‐tolerance in laboratory and field environmental gradient experiments, (c) experimental crosses, and (d) a candidate gene region approach for linkage mapping.

Two reviews (Kliebenstein, 2016; Züst & Agrawal, 2017) focused on possible mechanisms for the “grow‐or‐defend” trade‐off in plants, which most closely resembles the trade‐off detected here. They discussed (a) resource “flux” costs of resistance, (b) evidence for TFs from small‐sized Arabidopsis defense mutants, (c) correlated ecological costs of resistance and coordination between development and defense expression, and (d) pleiotropy or linkage disequilibrium in general. Using crosses and forward genetics, we found molecular evidence for pleiotropy that could have a physiological basis in signaling networks.

Other recent forward genetic studies have provided molecular evidence for pleiotropy in grow‐or‐defend trade‐offs. Genetic linkage analysis can identify pleiotropic regions of the genome when these traits comap (Anderson & Mitchell‐Olds, 2011). And genome‐wide association studies (GWAS) based on linkage disequilibrium can identify pleiotropic genes, as was recently shown for MYC1 in an analysis involving plant weight changes when plants of Arabidopsis faced challenges from both herbivory and drought (Davila Olivas et al., 2017).

In conclusion, our results indicating that regulation of GS could contribute to the evolution of the spatially restricted distributions are counter to previous hypotheses on defense evolution that have essentially argued the opposite, that variation in defensive chemistry is the consequence of spatial distributions, life history patterns etc. (Stamp, 2003). However, we do not know whether the trade‐off is widespread, and whether it is based on the co‐option of well‐documented antagonistic cross talk between signaling pathways.

## CONFLICT OF INTEREST

None declared.

## AUTHOR CONTRIBUTIONS

JO, GSG, RH, JA, and DHS carried out laboratory and field experiments; SLM handled carbon isotope ratio analysis; DHS conducted glucosinolate and statistical analysis and performed the crosses; JO and DHS wrote the manuscript; all coauthors read and commented on various written and oral versions of the study.

## Data Availability

All the data used for the results in this study are available in a public repository (https://doi.org/10.5061/dryad.k055k33).
