# Supplementary figures and images for: Evolutionary constraint on low elevation range expansion: Defense‐abiotic stress‐tolerance trade‐off in crosses of the ecological model Boechera stricta
Source: Ecol Evol. 2019 Oct 2;9(20):11532–44. doi: 10.1002/ece3.5499 (PMC6822064; doi:10.1002/ece3.5499)

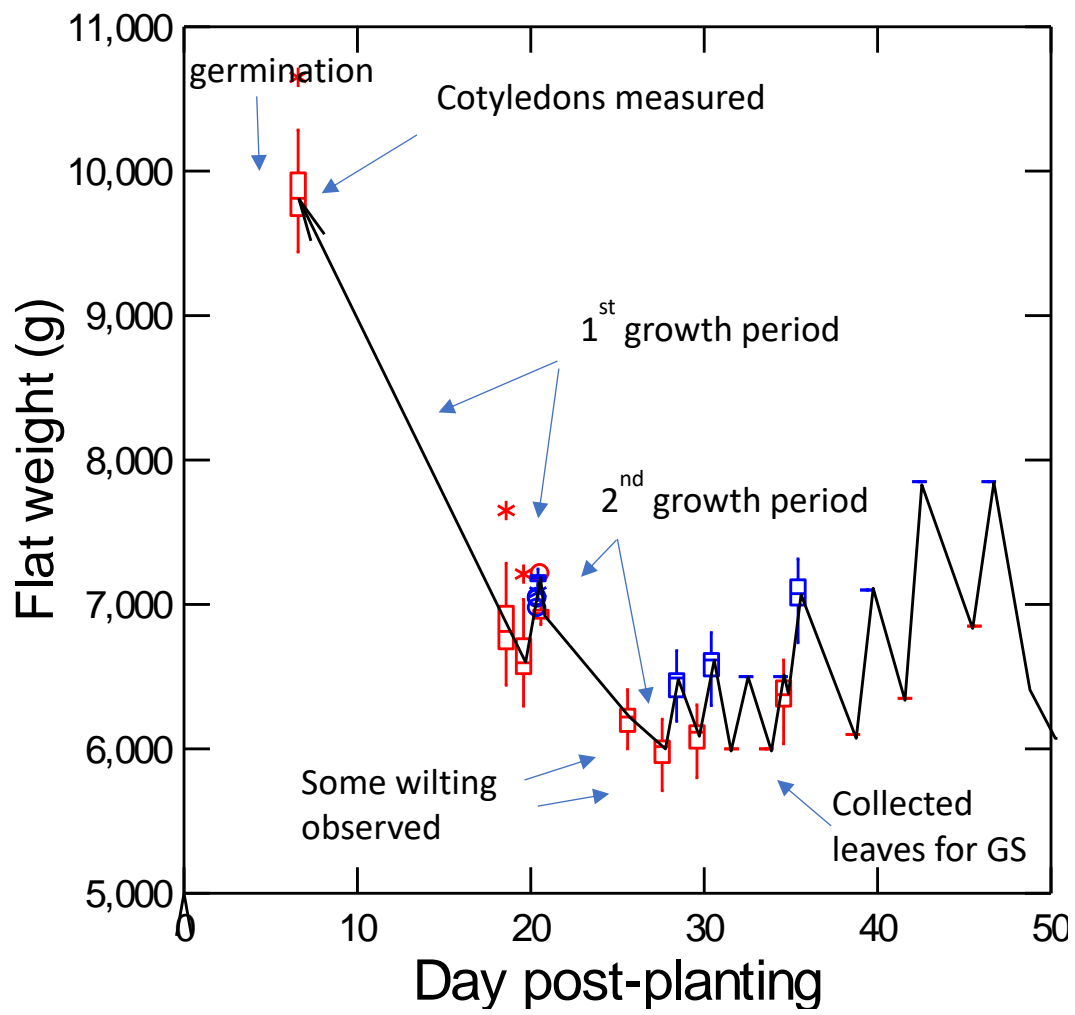

Supplement: Supplementary file 1 [file ECE3-9-11532-s001.pdf]

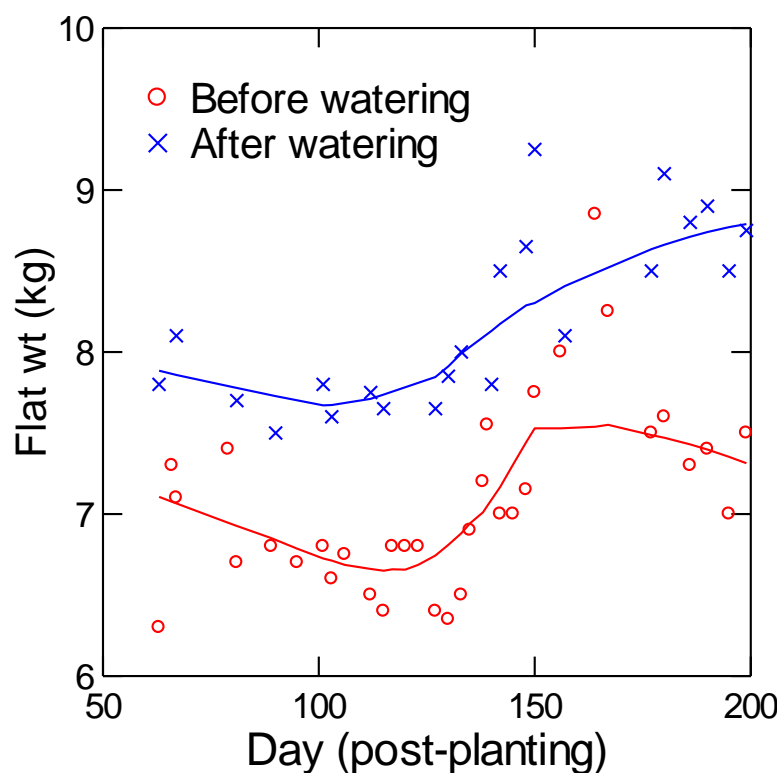

Supplement: Supplementary file 2 [file ECE3-9-11532-s002.pdf]

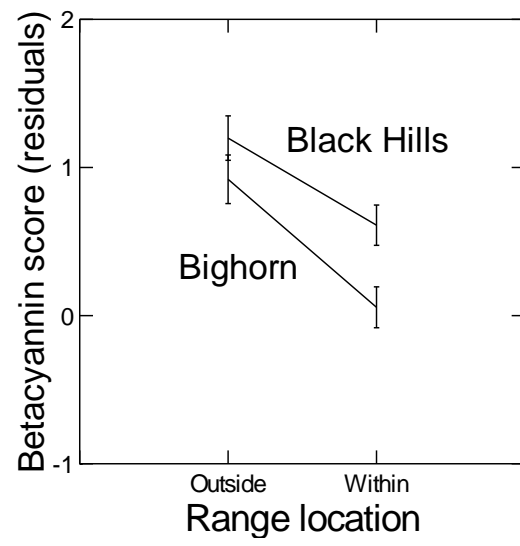

Supplement: Supplementary file 3 [file ECE3-9-11532-s003.pdf]

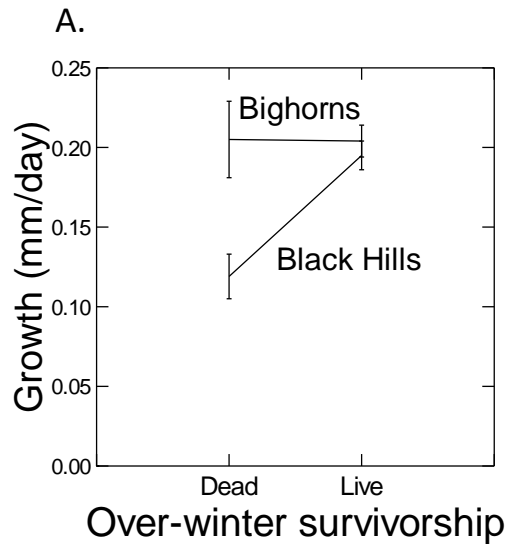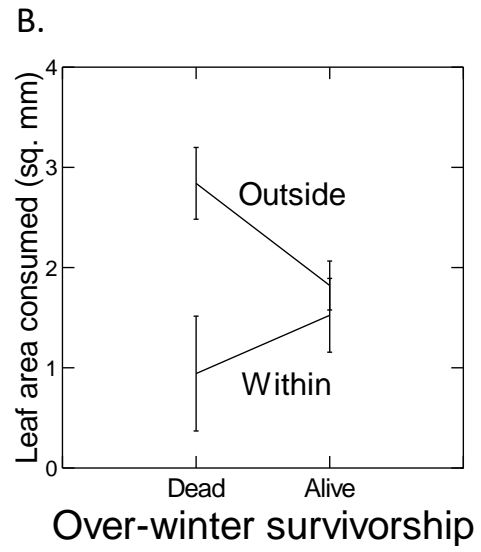

Supplement: Supplementary file 4 [file ECE3-9-11532-s004.pdf]

A.

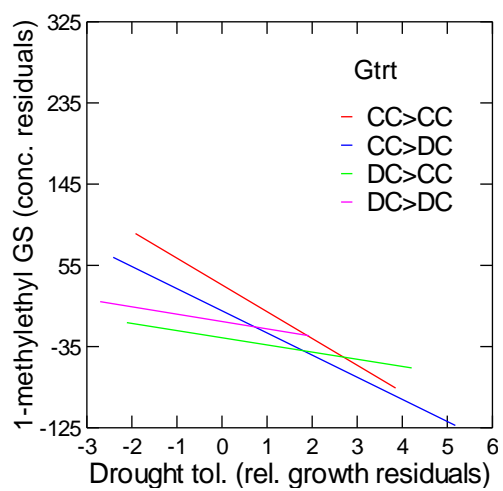

B.

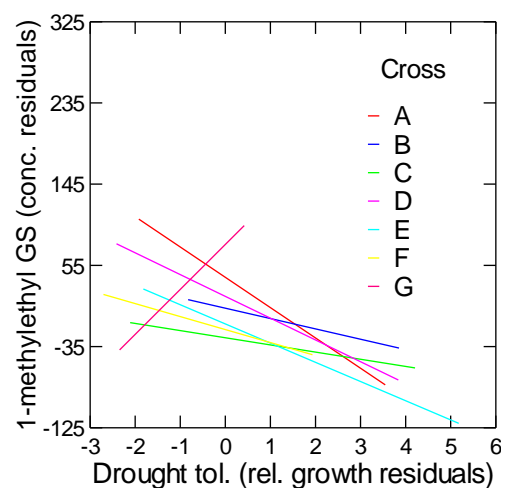

Supplement: Supplementary file 5 [file ECE3-9-11532-s005.pdf]

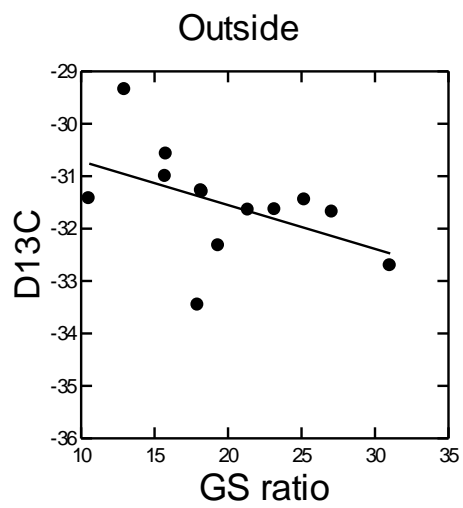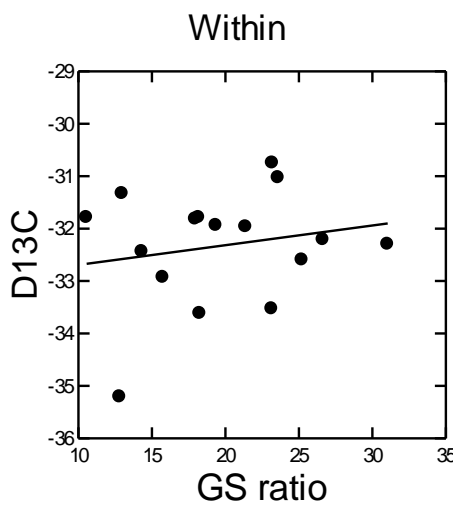

Supplement: Supplementary file 6 [file ECE3-9-11532-s006.pdf]
