# Supplementary material for: Evolutionary constraint on low elevation range expansion: Defense‐abiotic stress‐tolerance trade‐off in crosses of the ecological model Boechera stricta
Source: Ecol Evol. 2019 Oct 2;9(20):11532–44. doi: 10.1002/ece3.5499 (PMC6822064; doi:10.1002/ece3.5499)
Supplement: Supplementary file 7 [file ECE3-9-11532-s007.docx]

Table S1. Crosses conducted between Big Horn Mountain and Black Hills populations. Numbers represent inbred lines, but not necessarily the same individual plants. Arrow indicates the direction of pollen transfer. Recipient flowers were emasculated before opening and before anthers were matured. Donor stamens were brushed over recipient stigma surfaces. Breeding system can vary among and within *Boechera* species, although *B. stricta* is a diploid, sexual, predominantly inbreeding monophyletic species (Dobeš et al. 2004). Nonetheless, pollen shape from self-fertilized F1 plants was checked under a light microscope to determine breeding system. Pollen of F1 hybrids was uniformly elliptical, indicating a sexual breeding system (Beck et al. 2012) and therefore the segregation of alleles in the F2 generation. The parent plants were part of another experiment (Alsdurf et al. 2013, Alsdurf et al. 2015) that were watered differentially (control watered, CC, or drought stressed, DC, during basal rosette stage of life cycle. See Table S2). In the statistical analyses of the crosses in the present study we use type of cross (CC ↔ CC, DC ↔ CC, DC ↔ CC), noted by the symbol Gtrt for crosses or just Gtrt, without regard to direction of cross, to control for any variation due to these watering treatments two generations before. To avoid potential confounding effects, we also controlled for the different crosses (A to G) in separate analysis.

| Big Horn Mountains/Black Hills | Parent treatments | Cross name |
| --- | --- | --- |
| 63 🡪 48 | CC 🡪 CC | A |
| 60 🡨 48 | CC 🡨 CC | B |
| 61 🡪 47 | DC 🡪 CC | C |
| 63 🡪 48 | CC 🡪 DC | D |
| 62 🡨 47 | CC 🡨 DC | E |
| 61 🡪 46 | DC 🡪 DC | F |
| 63 🡨 51 | DC 🡨 DC | G |

Table S2. Inbred lines from high elevation Big Horn and low elevation Black Hills populations used in the Parent Line experiments. The naturally occurring inbred lines had been self-fertilized an additional 2-3 generations in the lab. Two generations before, the grandparents were part of another experiment (Alsdurf et al. 2013, Alsdurf et al. 2015) that were watered differentially (CC = control watering, DC = drought stressed), which here we call the grandparent treatments (Gtrt for lines or just Grt). In the grandparent experiment, the drought (D) treatment only occurred during the basal rosette stage of the life cycle; both CC and DC treatments were watered as controls during the reproductive stage of the life cycle, hence the second “C” used for both CC (control) and DC (drought) treatments. Variation in Gtrt here in the Parent Line experiments was confounded some by line; therefore, any variation in Gtrt may also be attributed some to variation among lines. In the present study, we used Gtrt and Line in separate analyses to help control for some variation within populations without confounding differences among populations.

| Population | Line | Gtrt |
| --- | --- | --- |
| Big Horn | 60 | CC |
| Big Horn | 61 | DC |
| Big Horn | 61 | DC |
| Big Horn | 62 | DC |
| Big Horn | 63 | CC |
| Big Horn | 63 | CC |
| Big Horn | 63 | DC |
| Black Hills | 46 | DC |
| Black Hills | 47 | CC |
| Black Hills | 47 | DC |
| Black Hills | 48 | CC |
| Black Hills | 48 | DC |
| Black Hills | 48 | DC |
| Black Hills | 51 | DC |

Table S3. F-ratios from ANCOVAs on glucosinolate (GS) production variables to determine differences between high and low elevation populations (see also Table 1 for population mean values). GS production variables: BCGS1 is 2-hydroxyl-1-methylethyl GS, BCGS2 is 1-methylethyl GS, METGS is 6-methylsulfinylhexyl GS, GS Ratio is (BCGS1 + BCGS2)/METGS. Because of high multi-colinearity among measures GS variables, MANCOVA was not possible. Effect of interest is Population (high elevation Big Horn vs. low elevation Black Hills). Other factors in the analysis controlled for other sources of variation. Gtrt is watering treatments of grandparents that were part of another study and that were confounded here by some variation among lines within populations (Table S2). Seedling size controlled for any developmental differences.

| Source | df | Total GS | BCGS1 | BCGS2 | METGS | GS Ratio |
| --- | --- | --- | --- | --- | --- | --- |
| Population | 1 | 15.367*** | 18.001*** | 11.165*** | 3.850* | 17.276*** |
| Gtrt | 1 | 7.611 | 9.081** | 6.469* | 0.341 | 18.903*** |
| Flat | 1 | 2.499 | 1.790 | 3.356* | 2.106 | 1.440 |
| Pop x Gtrt | 1 | 1.313 | 1.792 | 2.035 | 0.313 | 5.120* |
| Seedling size | 1 | 10.461** | 14.143*** | 0.908 | 10.979** | 0.358 |
| error | 67 |  |  |  |  |  |
| r^2^ |  | 38.3% | 40.5% | 34.7% | 36.4% | 55.7% |

*P < 0.05, **P < 0.01, ***P < 0.001

Table S4. F-ratios from MANCOVA and ANCOVAs on drought-stress tolerance traits to determine differences between high and low elevation populations (see also Table 1 for population mean values). Drought stress tolerance traits: leaf mass per area (LMA), relative growth (RELG), and wet and dry root:shoot ratio (Wet R:S and Dry R:S). Significance in MANCOVA protects univariate tests from Type I errors occurring from multiple testing. Effect of interest is Population (high elevation Big Horn vs. low elevation Black Hills). Other factors in the analysis controlled for other sources of variation. Gtrt is watering treatments of grandparents that were part of another study and that were confounded here by some variation among lines within populations (Table S2). Seedling size controlled for any developmental differences.

| Source | df | LMA | RELG | Wet R:S | Dry R:S | MANCOVA |
| --- | --- | --- | --- | --- | --- | --- |
| Population | 1 | 4.619* | 17.568*** | 28.168*** | 9.986** | 9.428*** |
| Gtrt | 1 | 1.857 | 1.430 | 5.548* | 5.519* | 1.972 |
| Flat | 1 | 0.218 | 1.833 | 4.114* | 4.041* | 12.135** |
| Pop x Gtrt | 1 | 0.072 | 3.035 | 2.416 | 0.766 | 1.053 |
| Seedling size | 1 | 67.046*** | 27.869*** | 1.126 | 1.28 | 14.665*** |
| error | 54 |  |  |  |  |  |
| r^2^ |  | 27.3% | 43.3% | 52.0% | 42.3% |  |

*P < 0.05, **P < 0.01, ***P < 0.001

Table S5. ANCOVA comparing high and low elevation populations for Betacyannin color score in field experiment across the low elevation range boundary at two sites. See Figure S3. r^2^ = 26.6%. Betacyannin increase indicates abiotic stress.

| Source | df | MS | F | P |
| --- | --- | --- | --- | --- |
| Population | 1 | 3.783 | 6.646 | **0.011** |
| Boundary | 1 | 14.479 | 25.435 | **<0.001** |
| Site | 1 | 4.276 | 7.511 | 0.007 |
| Seedling size | 1 | 0.247 | 0.433 | 0.512 |
| Plant size | 1 | 2.033 | 3.572 | 0.061 |
| Pop-by-Boundary | 1 | 0.535 | 0.940 | 0.334 |
| Error | 112 | 0.569 |  |  |

Table S6. ANCOVA comparing high and low elevation populations for performance (relative growth rate of basal rosettes) in field experiment across the low elevation range boundary at two sites. Differences in this measure of performance across the range were used to quantify abiotic stress tolerance. See Figure 2b for visual inspection of the significant Population-by-Range Boundary interaction. r^2^ = 54.8%.

| Source | df | MS | F | P |
| --- | --- | --- | --- | --- |
| Population | 1 | 1.760 | 1.421 | 0.236 |
| Boundary | 1 | 0.107 | 0.087 | 0.769 |
| Site | 1 | 0.053 | 0.043 | 0.837 |
| Seedling size | 1 | 6.786 | 5.482 | 0.021 |
| Plant size | 1 | 129.185 | 104.359 | 0.000 |
| Pop-by-Boundary | 1 | 9.445 | 7.630 | **0.007** |
| Error | 114 | 1.238 |  |  |

Table S7. ANCOVA for the relationship between growth rate (response variable) and overwinter survivorship. Analysis was conducted on plants that had not been fed upon by herbivores in the late fall census. Consequently, the overwinter mortality was caused mainly by abiotic stressors. Visual inspection of the marginally significant (P < 0.01) three-way interaction (Figure S4) indicated that low survivorship was associated with lower relative growth just across the low elevation range boundary for plants from the low elevation population.

| Source | df | MS | F | P |
| --- | --- | --- | --- | --- |
| Population | 1 | 0.005 | 3.058 | 0.083 |
| Boundary | 1 | 0.000 | 0.126 | 0.724 |
| Survivorship | 1 | 0.015 | 9.182 | **0.003** |
| Population-by-Boundary | 1 | 0.017 | 10.348 | **0.002** |
| Boundary-by-Survivorship | 1 | 0.002 | 1.142 | 0.288 |
| Population-by-Survivorship | 1 | 0.009 | 5.436 | **0.022** |
| Pop-by-Boundary-by-Survivorship | 1 | 0.006 | 3.376 | **0.069** |
| Seedling size | 1 | 0.004 | 2.527 | 0.115 |
| Plant size | 1 | 0.204 | 121.738 | 0.000 |
| Error | 111 | 0.002 |  |  |

Table S8. Amount of leaf area consumed by herbivores as a function of high and low elevation populations (Population), low elevation range boundary (Boundary), and over-winter survivorship (Survivorship). Whether the amount of leaf area consumed was not (A) or was (B) log transformed to better satisfy assumptions of normality for ANCOVA essentially did not affect the main result that damage was higher outside the range and that damage was associated with lower survivorship (Figure S4b).

| Source | df | MS | F | P |
| --- | --- | --- | --- | --- |
| Population | 1 | 0.002 | 0.000 | 0.995 |
| Boundary | 1 | 978.595 | 22.334 | **0.000** |
| Survivorship | 1 | 289.872 | 6.616 | **0.011** |
| Population-by-Boundary | 1 | 2.315 | 0.053 | 0.819 |
| Boundary-by-Survivorship | 1 | 311.214 | 7.103 | **0.009** |
| Population-by-Survivorship | 1 | 144.577 | 3.300 | 0.071 |
| Pop-by-Boundary-by-Survivorship | 1 | 22.029 | 0.503 | 0.479 |
| Seedling size | 1 | 2.800 | 0.064 | 0.801 |
| Plant size | 1 | 0.066 | 0.002 | 0.969 |
| Error | 141 | 43.816 |  |  |

r^2^ = 20.5%.

B.

| Source | df | MS | F | P |
| --- | --- | --- | --- | --- |
| Population | 1 | 1.590 | 1.574 | 0.219 |
| Boundary | 1 | 7.496 | 7.417 | **0.010** |
| Survivorship | 1 | 0.291 | 0.288 | 0.595 |
| Population-by-Boundary | 1 | 0.702 | 0.694 | 0.411 |
| Boundary-by-Survivorship | 1 | 4.048 | 4.005 | **0.054** |
| Population-by-Survivorship | 1 | 1.577 | 1.561 | 0.220 |
| Pop-by-Boundary-by-Survivorship | 1 | 0.239 | 0.236 | 0.630 |
| Seedling size | 1 | 0.675 | 0.668 | 0.420 |
| Plant size | 1 | 1.419 | 1.404 | 0.245 |
| Error | 33 | 1.011 |  |  |

r^2^ = 30.0%

Table S9. Several indicators of the tradeoff between abiotic stress tolerance and chemical defense. F-ratios from univariate analyses for effects of GS production variables on each of the three drought stress tolerance traits RELG, LMA and R:S (see Tables S3 and S4 for explanation of these abbreviations). Each test was protected from Type I statistical errors because of prior significance in multivariate analysis (Table 2) (Montgomery 1997). For each F, the df = 1, 494. In some cases, the effect of GS production on drought stress tolerance was dependent on Cross (as indicated significant Cross-by-GS interaction) (see Figure S5 for examples of the interactions detected). We conducted similar multivariate and univariate analyses controlling for Gtrt instead of Cross (Table S10). Gtrt, an inherited environmental effect, was confounded with Cross (see Table S1 for explanation of Gtrt).

|  | Total GS | BCGS1 | BCGS2 | METGS | GS Ratio |
| --- | --- | --- | --- | --- | --- |
| RELG | 7.222***^‡^ | 1.872^‡^ | 4.157*^‡^ | 0.131^‡^ | 6.091** |
| LMA | 4.52**^‡^ | 33.336***^‡^ | 87.809***^‡^ | 50.078***^‡^ | 11.017***^‡^ |
| R:S | 0.368 | 0.712^‡^ | 0.604 | 0.131^‡^ | 0.998 |

*P < 0.05, **P < 0.01, ***P < 0.001

^‡^Interaction with Cross was significant (Ps < 0.05).

Table S10. Several indicators of the tradeoff between abiotic stress tolerance and chemical defense controlling for Gtrt instead of Cross (see Tables 2 and S9 for the analyses that controlled for Cross; and see Table S1 for explanation of Gtrt, an inherited environmental factor that was confounded with Cross). The multivariate analysis (A) included all three abiotic stress tolerance variables RELG, LMA and R:S (see Tables S3 and S4 for explanation of these abbreviations), and the univariate (B) protected from Type I errors (Montgomery 1997). In the univariate analysis, F-ratios are shown for effects of GS production variables on each of the three drought stress tolerance traits. In some cases, the effect of GS production on drought stress tolerance was dependent on Gtrt (as indicated significant Gtrt-by-GS interaction).

A.

| Source | df | Total GS | BCGS1 | BCGS2 | METGS | GS Ratio |
| --- | --- | --- | --- | --- | --- | --- |
| GS | 3, 532 | 11.405*** | 6.835*** | 3.592*** | 11.392*** | 4.235** |
| Gtrt | 9, 46 | 2.686* | 1.040 | 0.660 | 0.975 | 1.399 |
| GS x Gtrt | 9, 1294 | 2.427** | 4.845*** | 2.196* | 2.859** | 2.022* |
| Flat | 63, 1588 | 5.609*** | 5.221*** | 5.378*** | 5.309*** | 4.474*** |
| Seedling size | 6, 1058 | 14.965*** | 16.668*** | 6.513*** | 19.740*** | 5.091*** |

*P < 0.05, **P < 0.01, ***P < 0.001

B.

|  | Total GS | BCGS1 | BCGS2 | METGS | GS Ratio |
| --- | --- | --- | --- | --- | --- |
| RELG | 11.3***^‡^ | .0.608^‡^ | 4.858*^‡^ | 1.110 | 4.352* |
| LMA | 12.128*** | 15.664***^‡^ | 85.273*** | 32.99***^‡^ | 10.767*** |
| R:S | 0.724 | 1.044^‡^ | 0.023 | 2.403 | 0.646 |

*P < 0.05, **P < 0.01, ***P < 0.001

^‡^Interaction with Gtrt was significant (Ps < 0.05).

Literature cited in supplementary tables:

Alsdurf, J., C. Anderson, and D. H. Siemens. 2015. Epigenetics of drought-induced trans-generational plasticity; consequences for range limit development. AoB Plants.

Alsdurf, J. D., T. J. Ripley, S. L. Matzner, and D. H. Siemens. 2013. Drought-induced trans-generational tradeoff between stress tolerance and defence: consequences for range limits? AoB Plants **5**:plt038.

Beck, J. B., P. J. Alexander, L. Allphin, I. A. Al-Shehbaz, C. Rushworth, C. D. Bailey, and M. D. Windham. 2012. Does hybridization drive the transition to asexuality in diploid *Boechera*? Evolution; international journal of organic evolution **66**:985-995.

Dobeš, C., S. T. Mitchell-Olds, and M. A. Koch. 2004. Intraspecific diversification in North American *Boechera stricta* (*Arabis drummondii*), *Boechera divaricarpa*, and *Boechera holboellii* (Brassicaceae) inferred from nuclear and chloroplast molecular markers – an integrative approach. American Journal of Botany **91**:2087-2101.

Montgomery, D. C. 1997. Design and analysis of experiments. 4th edition. John Wiley and Sons, New York.
